# HIV treatment as prevention — human rights issues

**DOI:** 10.1186/1758-2652-13-S4-O15

**Published:** 2010-11-08

**Authors:** J Amon

**Affiliations:** 1 Human Rights Watch, 350 Fifth Avenue, 34th Floor, New York, USA

## 

Recognizing access to HIV treatment as a human right has been important in efforts to ensure greater resources for the scale up of ART globally. However, it has not always been accepted by governments, and those asserting the right to HIV treatment have sometimes been in conflict with those asserting other rights, such as the right to intellectual property. Some governments have disputed their obligations to provide universal ART because of limited resources or competing priorities, while others have - in violation of human rights principles of non-discrimination and equality - challenged their obligation to provide it on an equitable basis to all within their borders, leaving out specific, often socially marginalized, groups. Understanding how HIV treatment can be successful as prevention will first require attention to these neglected issues. In addition, one of the greatest barriers to access to HIV treatment when available has been the failure of governments to protect individuals from HIV-related stigma and discrimination, which affects the willingness of individuals to be tested for HIV, to seek treatment if found to be positive and to adhere to medicines if they are attained. HIV treatment as prevention will require significantly greater efforts to address stigma and discrimination, and the promotion of truly voluntary HIV testing as a gateway to prevention and treatment, maintaining an emphasis on appropriate counseling, informed consent and confidentiality. The increasing popularity of HIV legislation that criminalizes intentional or attempted HIV transmission, and which sometimes has no exception for HIV-positive pregnant women unable to access PMTCT programs or individuals on ART with undetectable viremia, raises new challenges to expanding HIV testing and treatment programs and should be forcefully challenged by both clinical providers and human rights advocates.

